# Flagellin as a Versatile Adjuvant Platform: Genetic Fusion Approaches for Next-Generation Vaccines

**DOI:** 10.3390/ijms262110295

**Published:** 2025-10-22

**Authors:** Eugenia S. Mardanova, Nikolai V. Ravin

**Affiliations:** Research Center of Biotechnology of the Russian Academy of Sciences, 119071 Moscow, Russia

**Keywords:** flagellin, toll-like receptor 5, adjuvant, antigen, vaccine

## Abstract

Flagellin is the main structural protein of the bacterial flagellum, responsible for the movement of flagellated bacteria. Flagellin activates Toll-like receptor 5, inducing both innate and adaptive immune reactions, which highlights its potential as a vaccine adjuvant, particularly efficient in case of administration via mucosal routes. Genetic fusion of an antigen to flagellin has been shown to enhance the immune responses against the antigen. The molecular architecture of flagellin provides versatile and robust adjuvant functionality, facilitating the development of diverse vaccination strategies against multiple diseases as recombinant protein-based vaccines demonstrate substantial advantages over conventional live-attenuated and inactivated vaccines in both developmental efficiency and safety profiles. We present a comprehensive overview of vaccine design strategies employing genetic fusion of antigens to flagellin for protection against various infectious diseases. The proven effectiveness of flagellin-based delivery has enabled several vaccine candidates to enter clinical trials.

## 1. Introduction

Flagellin serves as the primary structural component of bacterial flagella, enabling motility in flagellated bacteria. As flagella represent an evolutionarily conserved and essential bacterial organelle, the immune system has adapted to recognize flagellin monomers as pathogen-associated molecular patterns (PAMPs), signaling potential bacterial infection [1].

**Figure 1:**
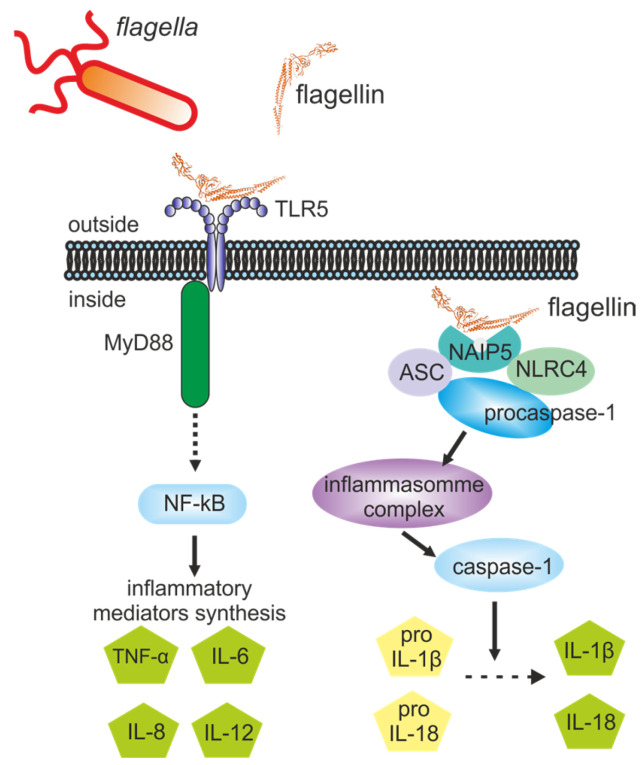
The TLR5-dependent NF-κB pathway, induced by extracellular flagellin, and the NLRC4/NAIP5-dependent inflammasome pathway, activated by cytosolic flagellin. MyD88, Myeloid Differentiation primary response 88 protein; NF-κB, Nuclear Factor kappa-light-chain-enhancer of activated B cells; TNFα, Tumor Necrosis Factor alpha; IL, interleukins; NAIP5, NLR family, neuronal apoptosis inhibitory protein 5; NLRC4, NLR family CARD-containing protein 4; ASC, apoptosis-associated speck-like protein containing a CARD (Caspase Activation and Recruitment Domain).

As crucial components of the innate immune system, Toll-like receptors (TLRs) and the Nucleotide-binding and oligomerization domain (NOD)-like receptors represent two major families of pattern recognition receptors that mediate pathogen detection and initiate innate immune responses [2]. Flagellin stimulates both TLR5 and cytosolic NOD-like receptor (NLR) to coordinate adaptive immunity against pathogens (Figure 1).

The activation of TLR5 signaling involves the formation of a tail-to-tail 2:2 complex composed of two TLR5-flagellin heterodimers, where stability is provided by quaternary contacts of the N-terminus of flagellin with the convex surface of the opposing TLR5 [3]. This engagement triggers recruitment of the TIR-domain adaptor MyD88 (Myeloid Differentiation primary response 88 protein), initiating the IRAK (Interleukin-1 Receptor-Associated Kinase)–TRAF6 (TNF Receptor-Associated Factor 6)–TAK1 (TGF-β-Activated Kinase 1)–NFκB (Nuclear Factor kappa-light-chain-enhancer of activated B cells) signaling axis and subsequent production of proinflammatory cytokines such as TNFα (Tumor Necrosis Factor alpha) and interleukins IL-6, IL-8, and IL-12 [4,5].

Epithelial cells in mucosal linings (respiratory, urogenital, renal, and intestinal tracts) express TLR5 extensively, positioning them as frontline defenders against pathogens. This, combined with the presence of TLR5 on dendritic cells and macrophages, highlights the suitability of flagellin as a mucosal adjuvant [6]. Dendritic cells, T-cells, and B-cells among these recruited populations then couple a powerful innate immune reaction with adaptive immunity [7]. Administering flagellin via mucosal routes elicits strong dual immunity, stimulating both mucosal IgA production and systemic IgG responses.

Activation of NLRs by cytosolic flagellin involves two specialized NLR: NAIP5 (NLR family, neuronal apoptosis inhibitory protein 5) and NLRC4 (NLR family CARD-containing protein 4) [8,9,10]. Flagellin-NAIP5 binding results in interaction of NAIP5 with NLRC4 and subsequent NLRC4 activation [11]. Bacterial flagellin triggers activation of the NLRC4 inflammasome through a specific mechanism: its C-terminal helical domain binds to a narrow pocket in the NAIP5 receptor [12]. This recognition initiates the assembly of a hetero-oligomeric NAIP5/NLRC4 inflammasome complex, and recruit procaspase-1 via caspase-activating and recruitment domain (CARD) of NLRC4 or the adaptor protein ASC (apoptosis-associated speck-like protein containing CARD), which binds to inflammasome during inflammasome complex formation. The later activates caspase-1. Activated caspase-1 then mediates proteolytic cleavage of pro-IL-1β and pro-IL-18 to their mature forms, ultimately leading to pyroptotic death of macrophages and the release of IL-1β [13,14].

Following the initial activation of TLRs, the adaptive immune response unfolds: antigen-presenting cells direct CD4+ T cell differentiation into Th1 (driven by TNF-α, IL-12, IFNs) or Th17 (driven by IL-1, IL-6, IL-23) subsets. Th1 cells produce IFN-γ, while Th17 cells produce IL-17. This phase also expands antigen-specific CD8+ T cells and activates B cells resulting in the production of antigen-specific antibodies [15].

Flagellin has been shown to induce a Th1 profile in CD4+ T-cells from both newborns and adults, notably overcoming the epigenetic suppression of IL-12 and IFN-γ characteristic of early life. Furthermore, flagellin treatment has been shown to alter the Th1/Th2 profile of natural memory cells in adult blood, reducing IL-4 expression by Th2 cells and increasing IFN-γ production by the Th1 population [16].

The production of specific IgG isotypes serves as a proxy for T-helper cell polarization, with IgG2a and IgG1 being induced by Th1 (via IFN-γ) and Th2 (via IL-4) responses, respectively [17]. The direction of the immune response (Th1 or Th2) to *S. typhimurium* flagellin FliC depends entirely on the method of its presentation to the immune system. The soluble form of FliC induces a Th2 response, as evidenced by the production of IL-4 and IgG1 antibodies. Conversely, when FliC was presented in its natural form on the surface of live salmonella, a Th1 response is induced, with the production of IFN-γ and IgG2a antibodies [18]. In our previous studies, we used *S. typhimurium* flagellin as a scaffold to present the M2e peptide of the influenza A virus. In a murine model, immunization with these fusion protein predominantly induced IgG1 rather than IgG2a type antibodies against M2e, indicating a Th2-polarized immune response [19,20]. These data convincingly demonstrate that it is not the intrinsic properties of the antigen, but the context of its interaction with the immune system, that determines T-helper cell polarization.

## 2. Three-Dimensional Structure of Flagellin and Its Domain Organization

**Figure 2:**
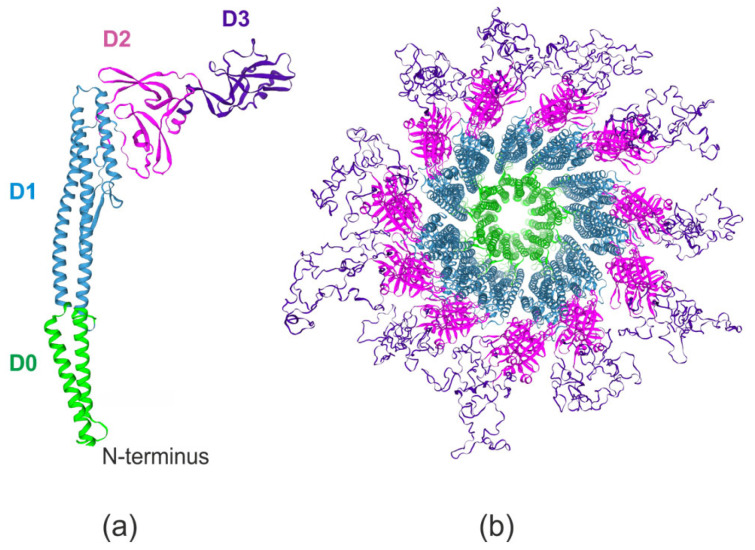
The 3D structure of *S. typhimurium* flagellin FljB (PDB ID: 1UCU): D0 domain (1–40, 455–494 aa); D1 domain (41–169, 400–454 aa); D2/D3 domains (170–399 aa). (**a**) Monomer structure; (**b**) Structure of homo-22-mer. The structure was predicted using SWISS-MODEL server [23].

The atomic-resolution structure of flagellin-TLR5 complex has been elucidated, establishing a structural basis for mechanistic studies of flagellin’s immunostimulatory properties [21]. The canonical flagellin structure (Figure 2a) exhibits a boomerang-shaped architecture composed of four distinct domains: D0 (N- and C-terminal α-helices), D1 (TLR5-binding domain), D2 (central linker), and D3 (hypervariable region) [22].

Comparative studies of flagellin homologs revealed that the D0/D1domains maintain high sequence conservation, while the D2/D3 domains show greater sequence variation [24]. Flagellin polymerization results in a filament architecture where the structurally conserved D0/D1 domains constitute the inner core and the more variable D2/D3 domains face the external environment (Figure 2b).

Crystallographic studies of truncated zebrafish TLR5 bound to flagellin identified three essential D1 domain segments (a.a. 82–101, 110–118, and 412–438) mediating the interaction. Among these, the a.a. 89–96 sequence emerged as the dominant TLR5 activation motif [25]. Structural and functional analyses reveal distinct roles for flagellin domains: D1 facilitates TLR5 interaction and dimerization, while D0 is indispensable for downstream signaling. Deletion of D0 results in three orders of magnitude signaling impairment without affecting receptor binding [26]. Current structural and functional evidence indicates that flagellin’s D2 and D3 domains are not involved in TLR5 activation [27]. A later investigation of flagellin derivatives derived from *Escherichia coli* (EHEC EDL933) revealed the essential role of the D0/D1 domains in TLR5 activation, which is important for the optimization of flagellin derivatives for vaccine development [28]. The C-terminal 35 a.a. of flagellin (within the D0 domain) are responsible for NAIP5-mediated inflammasome activation [4]. 

In summary, the D0 and D1 domains of flagellin are critical for innate immune recognition and flagellar filament polymerization, while the D2 and D3 domains contain primary antigenic epitopes and contribute to phase variations, allowing bacteria to evade the host immune response. This structural and functional separation allows flagellin to maintain its important role in motility while also adapting to the selective pressures of the immune system [27].

Knowledge of the structure of flagellin and the location of its functional domains opens up possibilities for the rational design of flagellin-based proteins for immunostimulatory purposes.

## 3. Flagellin as a Vaccine Adjuvant: Genetic Fusion Versus Co-Formulation

Numerous studies have investigated the potential of flagellin as a mucosal adjuvant [29]. Initially, the strategy of expression of chimeric genes or co-display of flagellin and foreign antigens in live bacterial strains was exploited [30]. Chimeric flagellin was constructed by inserting the coding sequences of foreign antigens into the highly variable region of flagellin. When such a fusion protein was exposed on the flagellum surface, normal flagellum structure and function were maintained.

Subsequently, the development of flagellin-based vaccines has mainly followed the strategy of producing purified recombinant proteins, since this type of vaccine provides advantages in terms of safety. Flagellin can be integrated into subunit vaccines either through simple co-formulation with antigens or through the creation of recombinant fusion proteins combining flagellin with antigen. Various experimental evidences showed that genetically engineered flagellin–antigen fusion proteins generate more consistent immunostimulation than physical mixtures [7]. Genetic attachment of flagellin to antigens enhances antigen presentation to APCs, reduces the required dose, and eliminates the need for additional adjuvants.

## 4. Recombinant Chimeric Proteins Combining Antigens with Flagellin Scaffolds

The domain organization of flagellin facilitates the incorporation of foreign peptide sequences into various sites, including the N- and C-terminal regions, as well as the internal hypervariable domain, without compromising protein function.

Development of flagellin-based vaccines has involved a variety of genetic fusion strategies using full-length flagellin constructs as well as structurally modified variants. The reduction in flagellin molecular weight results in smaller fusion constructs, which may facilitate protein expression and purification. Also, without the hypervariable region, less antibody response will be generated against the flagellin backbone [31]. A summary of flagellin–antigen fusion proteins is presented in Supplementary Table S1.

### 4.1. Proof-of Principle: Ovalbumin

**Figure 3:**
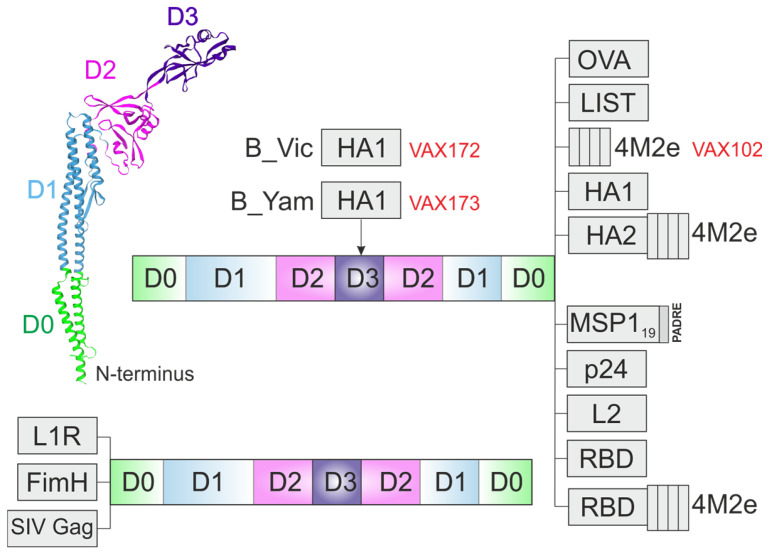
Full-length flagellin as an adjuvant. The structures of flagellin-based chimeric proteins are shown. OVA, ovalbumin; LIST, two epitopes derived from the *Listeria monocytogenes*; 4M2e, four tandem copies of the ectodomain of the influenza protein M2; HA1, the globular head domain of the influenza A hemagglutinin (HA); HA2, the stalk domain of HA; MSP1_19_, the *Plasmodium vivax* Merozoite Surface Protein-1; p24, HIV-1 p24 protein; L2, L2 protein of human papillomavirus; L1R, outer membrane protein antigen of the vaccinia virus; FimH, adhesion of uropathogenic *Escherichia coli*; SIV Gag, simian immunodeficiency virus (SIV) Gag protein; RBD, the receptor-binding domain of the SARS-CoV-2 spike glycoprotein; VAX, vaccine developed by VaxInnate Corp., Cranbury, NJ, USA.

One of the first recombinant flagellin-based proteins was chicken ovalbumin (OVA) linked to the C-terminus of *Salmonella typhimurium* flagellin FljB [32] (Figure 3). Chimeric protein was expressed in *E. coli*. The immunogenicity of purified recombinant proteins was examined in vivo. C57BL/6 mice were subcutaneously immunized with fusion flagellin-OVA protein, an equimolar dose of OVA alone or mixed with recombinant flagellin. OVA-specific IgG1 and IgG2a antibody responses were detected in the sera of mice immunized with the fusion protein, but not in mice immunized with an OVA alone or mixed with flagellin. These results indicated that flagellin fusion with antigen in a single protein is necessary to enhance the immunogenicity of the antigen. Furthermore, it was demonstrated that immune responses elicited to flagellin do not inhibit subsequent TLR5-mediated activation or the immune response to recombinant flagellin fusion proteins in vitro or in vivo. Flagellin fusion proteins were shown to induce antigen-specific T and B cell responses in vivo. Following the proven concept with ovalbumin, multiple antigens have subsequently been incorporated into flagellin-based constructs.

### 4.2. Flagellin as an Adjuvant for Recombinant Vaccines Against Influenza

Influenza is one of the most widespread infectious diseases, causing annual epidemics and periodic pandemics. Traditional influenza vaccines based on the major surface antigens, hemagglutinin and neuraminidase, are highly effective. However, due to the antigenic variability of these proteins, such vaccines are strain-specific and require almost annual updating [33]. An attractive alternative could be the development of a “universal” vaccine based on conserved influenza virus antigens [34]. However, such antigens are small in size and poorly immunogenic. Therefore, already in the first studies on the use of flagellin as an adjuvant, conserved peptide antigens of the influenza A virus were targeted.

A recombinant protein comprising flagellin fused to four tandem copies of the ectodomain of the conserved influenza A virus protein M2 (M2e) (Figure 3), obtained in *E. coli*, induced the production of M2e-specific antibodies and protected mice from a lethal challenge with influenza A virus [35]. Phase I/II clinical trials (NCT00921947, NCT00603811) subsequently evaluated the safety profile, reactogenicity, tolerability, and adverse event incidence of VAX102 vaccine (flagellin-4M2e fusion) administered via intramuscular or subcutaneous routes in healthy adult participants [36]. Clinical trials demonstrate that flagellin-M2e fusion proteins exhibit favorable safety and tolerability profiles while demonstrating efficacy against influenza A. This vaccine platform may provide long-lasting protection over several seasons without the need for seasonal reformulation.

The globular head domain (HA1) of the protective antigen hemagglutinin (HA) of A/Puerto Rico/8/34 (PR8) influenza virus was linked to the C-terminus of the *S. typhimurium* flagellin FljB [37] (Figure 3). In mouse models of influenza infection, the vaccines induced robust antibody production and protected mice from lethal challenge. The same design principle was implemented for the seasonal strain A/Solomon Islands/03/2006 with strong immunogenicity of the flagellin-HA1 fusion (VAX125) protein observed in both mice and rabbits. Clinical evaluation in phase I/II trials (NCT00730457) revealed that VAX125 exhibited favorable tolerability and immunogenicity profiles in humans [38].

**Figure 4:**
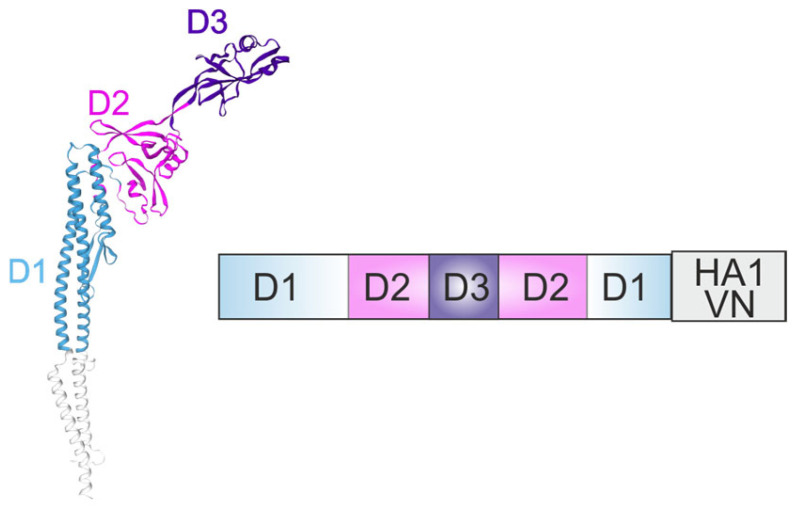
Flagellin lacking the D0 domain as an adjuvant. The structure of flagellin-based chimeric protein is shown. HA1(VN), the HA1 domain of influenza virus A/Vietnam/1203/04.

**Figure 5:**
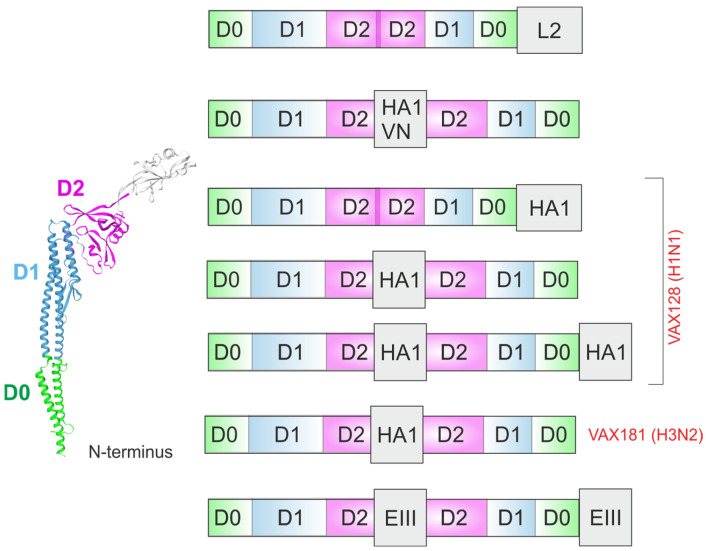
Flagellin lacking the D3 domain as an adjuvant. The structures of flagellin-based chimeric proteins are shown. L2, L2 protein (a.a. 11–200) of human papillomavirus (HPV16); HA1 (VN), the HA globular head domain of influenza virus A/Vietnam/1203/04; HA1, the HA globular head domain of influenza virus A/California/7/2009 (H1N1); EIII, the III domain of the dengue virus E protein; VAX, vaccine developed by VaxInnate Corp., USA.

Later, the HA1 domain of influenza virus A/Vietnam/1203/04 (VN) was fused to the C-terminus of the full-length *S. typhimurium* FljB protein (Figure 3) or to the C-terminus of D0-defficient flagellin (a.a. 1–46 and a.a. 465–506, HA1-2 fused after a.a. 464) (Figure 4), or in place of the D3 domain (a.a. 191–292) (Figure 5).

In the C-terminal fusion, the last amino acid of flagellin, R506, was mutated to A506 to reduce proteolytic cleavage [39]. Replacement of the D3 domain with HA1(VN) (Figure 5) was found to create a highly effective vaccine that elicited high titers of antibodies that protected mice from disease and death in a lethal challenge model. In contrast, the construct lacking the D0 domain (Figure 4) failed to elicit high levels of anti-HA antibodies in serum. These data demonstrate the importance of the D0 domain for the adjuvant activity of flagellin.

Exploiting the same strategy, a vaccine (VAX128) based on A/California/7/2009 (H1N1) strain was developed [40] (NCT01172054). The HA1 domain of the novel H1N1 virus was genetically fused to the C-terminus of flagellin (VAX128A) (Figure 3) or used to replace the D3 domain (VAX128B) (Figure 5) or fused at both positions (VAX128C) in the VAX128 vaccine (Figure 5). The replacement of the D3 domain with a C-terminal fusion enhanced both the safety profile and immunogenicity of VAX128C compared to the alternative constructs.

VAX2012Q represents a quadrivalent influenza vaccine composed of four HA-flagellin fusion proteins (Figure 5). The four proteins were VAX128C, VAX181 (H3N2 virus) (HA1 was inserted in place of the D3 domain), VAX173 (influenza B virus, Yamagata lineage), and VAX172 (influenza B virus, Victoria lineage) (HA1 was inserted in place of the D3 domain). The safety and immunogenicity of this vaccine at different doses were evaluated in phase 1/2 trials. In general, VAX2012Q elicited immune responses at all tested doses with no critical safety concerns [41].

Full-length flagellin of *S. typhimurium* fused to four tandem copies of the M2e peptide (Flg-4M) has also been expressed in *Nicotiana benthamiana* plants using a self-replicating vector based on potato virus X (PVX). Intranasal immunization of mice with purified Flg-4M protein induced high levels of M2e-specific serum antibodies and provided protection against lethal challenge with influenza virus [42]. The same approach was used for the development of a recombinant bivalent vaccine candidate against influenza A and COVID-19. The receptor-binding domain of the SARS-CoV-2 spike glycoprotein (RBD) alone or in combination with 4M2e was fused to C-terminus of flagellin (Figure 3). Plant-produced RBD and RBD-4M2e could be further used for the development of subunit vaccines against COVID-19 and a bivalent vaccine against COVID-19 and influenza A [43].

**Figure 6:**
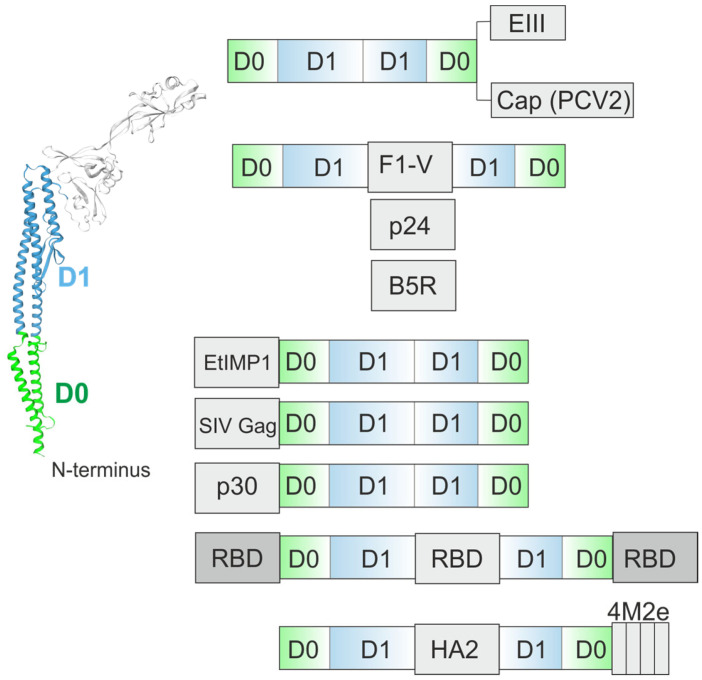
Flagellin lacking the D2/D3 domains as an adjuvant. The structures of flagellin-based chimeric proteins are shown. EIII, the III domain of the dengue virus E protein; Cap (PCV2), the porcine circovirus 2 (PCV2) capsid protein; F1-V, the fraction 1 capsular antigen (the F1 antigen) and LcrV (also termed the V antigen) of *Yersinia pestis*; p24, HIV-1 p24 protein; SIV Gag, simian immunodeficiency virus (SIV) Gag protein; B5R, outer membrane protein antigens B5R of the vaccinia virus; EtIMP1, immune mapped protein-1 (IMP1) of apicomplexan parasite *Eimeria tenella*; RBD, the receptor-binding domain of the SARS-CoV-2 spike glycoprotein; HA2, the stalk HA domain; 4M2e, four tandem copies of the ectodomain of the conserved influenza matrix protein M2; p30, the African swine fever virus protein p30.

Along with the use of the HA1 head domain, the HA2 stalk domain (conserved HA2 regions, a.a. 76–130) was also fused to flagellin in combination with four copies of M2e. HA2 and M2e peptides were linked to the C-terminus of full-length flagellin or HA2 domain (Figure 3) was inserted instead of the D2/D3 domain while 4M2e was linked to the C-terminus of modified flagellin (Figure 6). Recombinant proteins induced strong M2e-specific IgG response and CD4+/CD8+ T-cell response. The insertion of HA2 into the hypervariable domain of flagellin greatly increases antigen-specific T-cell response, while both proteins provided full protection of immunized mice against lethal influenza challenge [19,20]. Full-length flagellin comprising HA2 and 4M2e was also successfully expressed in *N. benthamiana* plants providing immunogenicity and protection potential in mice [44].

### 4.3. Flagellin-Based Vaccine Candidates Targeting Other Pathogens

Along with the development of recombinant influenza vaccines, the strategy of fusing antigen with flagellin to create a highly immunogenic vaccine composition has been used over the past two decades for various human and animal pathogens.

A fusion protein consisting of flagellin and a polypeptide containing two immunoprotective epitopes derived from *Listeria monocytogenes* antigens p60 and listeriolysin O was constructed (Figure 3). Animals immunized with this recombinant protein demonstrated significant antigen-specific CD8+ T cell responses and protection upon challenge with virulent *L. monocytogenes* [32]. Subsequently, the method of genetic fusion was used to link antigens of various infectious agents to full-size flagellin (Figure 3).

Similarly to the influenza VAX128 platform, the envelope protein domain III (EIII, residues 289–400) from the four dengue virus serotypes (DENV-1 strain 16007, DENV-2 strain 16681, DENV-3 strain Nicaragua 24/94, and DENV-4 strain 341750) was used to generate a tetravalent dengue vaccine (TDV). The vaccine comprises four distinct constructs. In each construct, two copies of a serotype-specific EIII domain were fused to the flagellin adjuvant: one at the C-terminus and one in place of the D3 domain (Figure 5). In animal models, the flagellin-EIII vaccine elicited durable neutralizing antibodies and provided partial protection in primates [45].

Malaria vaccine candidates based on the *Plasmodium vivax* Merozoite Surface Protein-1 (MSP1_19_) in combination with the Pan allelic HLA DR-binding epitope (PADRE) fused to the C-terminus of *S. typhimurium* FliC flagellin (Figure 3) elicited strong and long-lasting MSP1_19_-specific systemic antibody responses [46].

The adhesin FimH, one of the most important uropathogenic *E. coli* virulence factors, was fused to the N-terminus of *E. coli* flagellin (FliC) (Figure 3). Mice immunized with the fused FimH-FliC protein induced significantly higher markers of humoral (IgG1 and IgG2a) and cellular (IFN- and IL-4) immune responses than with FimH alone or FimH admixed with FliC [47].

The outer membrane protein antigens L1R and B5R of the vaccinia virus possess low immunogenicity. To increase the immunogenicity of this antigen, FliC flagellin from *S. enterica* serovar enteritidis and a truncated version of flagellin (a.a. 1–195, a.a. 379–505) were used. The L1R antigen was linked to the N-terminus of full-length flagellin (Figure 3). The B5R antigen was inserted in place of the hypervariable domain of flagellin (Figure 6). Flagellin-L1R and flagellin-B5R fusion proteins were more potent than flagellin, L1R, and B5R taken as separate proteins. Three immunizations with flagellin-L1R and flagellin-B5R fusion proteins were required to confer protection of mice against vaccinia virus challenge [48].

The L2 protein (a.a. 11–200) of human papillomavirus (HPV16) was linked to the C-terminus of full-length flagellin (Figure 3). Since the D3 domain is not required for TLR5 activation, this antigen was also fused to the C-terminus of a truncated version of flagellin lacking the D3 domain. Additionally, two versions of concatenated L2, representing either alpha-9 and alpha-7 species or all five oncogenic alpha species, were linked to the C-terminus of truncated flagellin lacking the D3 domain. Recombinant fusion proteins provided complete protection against infection (mice) and disease (rabbits) following challenge with homologous or heterologous HPV [49].

The EIII domain of the West Nile virus (WNV) envelope protein was linked to truncated flagellin of *S. typhimurium* lacking the hypervariable region spanning a.a. 170–415 (Figure 6). A truncated flagellin-based fusion protein produced in *E. coli* and purified under denaturing conditions elicited a strong VNV-E-specific immunoglobulin G antibody response that neutralized viral infectivity and provided protection against lethal WNV challenge [50].

The Cap protein of porcine circovirus type 2 (PCV2), a major host-protective immunogen, was linked to truncated flagellin of *S. typhimurium* (Figure 6) and produced in a baculovirus system. The expressed recombinant protein was purified under denaturing conditions. Experiments in laboratory animals showed that the flagellin-Cap fusion protein elicited a stronger PCV2-specific IgG antibody response, higher levels of neutralizing antibodies, and higher secretion of cytokines such as TNF-α and IFN-γ, providing better protection against viral challenge than mice administered with recombinant Cap alone [51].

The fraction 1 capsular antigen (the F1 antigen) and LcrV (also termed the V antigen) of *Yersinia pestis* were fused to flagellin (*S. enterica* FliC). A large part of the hypervariable domain of flagellin (a.a. 196–378) has been deleted, and the F1-V fragment was inserted (Figure 6). This fusion protein induces a strong antigen-specific antibody response in mice and two non-human primate species. Immunized mice showed complete protection when challenged intranasally 150 mean tolerated doses of *Y. pestis* CO92 [52]. Based on preclinical results, the safety, immunogenicity, and tolerability of the recombinant flagellin-F1-V fusion protein were evaluated in phase I clinical trials (ClinicalTrials.gov Identifier: NCT01381744).

A recombinant rhesus cytomegalovirus (RhCMV) was constructed expressing the Gag protein of simian immunodeficiency virus (SIV) in fusion with the FliC protein of *Salmonella*, in which the region containing immunodominant epitopes was deleted (Figure 6). Two FliC truncations were constructed by deleting the nucleotides encoding a.a. 196–378 or 141–398. Determination of the TLR5-specific activity of truncated FliC revealed the feasibility of a deletion variant of a.a. 196–378. The SIV Gag protein was linked to the N-terminus of full-length flagellin (Figure 3) or this truncated variant. The results showed that an engineered replicating RhCMV expressing the SIV Gag/FliC fusion protein was able to activate TLR5 in a macrophage cell line in vitro and induce an altered inflammatory response in vivo [53].

*E. coli* K-12 flagellin was used to present the HIV-1 p24 protein as a model object. This protein was linked to the C-terminus of full-length flagellin (Figure 3) or inserted in place of the D2/D3 domains (a.a. 172–405) (Figure 6). The truncated flagellin triggered much weaker systemic inflammation and showed no signs of causing inflammatory side effects in mice, even at higher doses, than the full-length variant. Thanks to its dual effect of increasing IgA levels and reducing inflammatory reactions, the truncated p24-flagellin fusion offers a wider therapeutic window, positioning it as a potential breakthrough in the development of mucosal adjuvants [54].

Flagellin FliC with a deleted hypervariable domain (180–400 a.a.) was used to present immune mapped protein-1 (IMP1) of the apicomplexan parasite *Eimeria tenella*. IMP1 was linked to the N-terminus of truncated FliC (Figure 6). Immunization of chickens with the recombinant fusion protein resulted in a stronger cellular immune response than immunization with recombinant IMP1 alone [55].

The African swine fever virus p30 protein (p30) was fused to the N-terminus of D2/D3-deficient *S. typhimurium* flagellin. Immunization with the recombinant fusion protein produced in *E. coli* significantly enhanced both humoral and cellular immune responses in mice compared to the antigen alone [56].

A chimeric triple-RBD immunogen, comprising one Delta-type RBD and two Omicron-type RBDs of SARS-CoV-2, was linked to the truncated flagellin (ΔD2/D3). RBD sequences were linked to the N- and C-termini and inserted instead of the D2/D3 domains simultaneously [57] (Figure 6). The study demonstrated that the RBD-flagellin fusion vaccine induced strong and broadly neutralizing IgG in plasma as well as IgA in salivary secretions [58].

Wang et al. [59] designed a novel flagellin-based adjuvant consisting of bacterial flagellin fused with nanobodies (Nb) specific to the PCV2 Cap protein (Nbcap-flagellin). The Nbcap-flagellin protein utilizes the ability of nanobodies to bind to the Cap protein, forming an antigen–adjuvant complex to enhance the immunogenicity of the vaccine. Vaccination of mice with PCV2-Cap and Nbcap-flagellin induced significantly higher levels of Cap-specific antibodies and immune-related cytokines compared to the PCV2-Cap without adjuvant and the mixture of Cap protein with flagellin.

## 5. N-Terminal Region of Flagellin as an Adjuvant

The above data demonstrate that the D0 domain is essential for flagellin’s immunostimulatory activity, and this activity was not impaired by deletion of the D2/D3 variable region. Several studies have tested the possibility of using only the N-terminal fragment containing the D0 or D0/D1 domains as an adjuvant [60,61,62,63,64,65,66].

**Figure 7:**
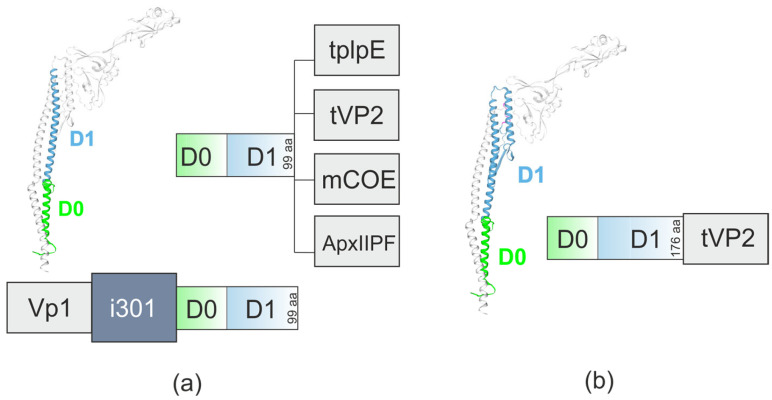
N-terminal flagellin D0/D1 domains as adjuvant. The structures of flagellin-based chimeric proteins comprising a.a. 1–99 (**a**) or a.a. 1–176 (**b**) of the D0/D1 domains are shown. tplpE, lipoprotein E of *P. multocida*; Vp1, protein of Foot-and-mouth disease; tVP2, truncated VP2 protein of the Infectious bursal disease virus; mCOE, modified core neutralizing epitope of the porcine epidemic diarrhea virus; ApxIIPF, the pore-forming region of the exotoxin ApxII of *A. pleuropneumoniae*; i301, self-assembling protein.

Candidate vaccine against fowl cholera, a bacterial disease caused by *Pasteurella multocida* serotype A, based on *P. multocida* lipoprotein E linked to the C-terminus of a truncated *S. typhimurium* FliC flagellin (only the first 99 a.a., Figure 7) induced a humoral immune response in chickens and increased survival to 75%, compared to 25% in the antigen-only group [63].

The truncated VP2 protein (tVP2, a.a. 199–356) of the Infectious bursal disease virus (IBDV) was genetically linked to the *S. enterica* flagellin FliC. Two truncated variants of FliC were used, namely a.a. 1–99 and a.a. 1–176 (Figure 7). Antigen of IBDV was linked to the C-terminus. Both antigen–adjuvant fusions elicited potent immune responses, including higher IgG levels, enhanced cellular immunity, and increased secretion of IL-4 and IFN-γ in comparison to tVP2 alone. Additionally, the produced antibodies effectively neutralized IBDV. Antibody and cytokine titers were not statistically different when using the two flagellin-VP2 proteins. However, neutralizing antibody titers were significantly higher in case of the longer FliC version [64].

A fusion protein vaccine, which combines the N-terminal region of flagellin (FliC, a.a. 1–99) with a modified core neutralizing epitope (mCOE) of the porcine epidemic diarrhea (PED) virus (PEDV) (Figure 7) effectively stimulated cellular immunity and neutralized PEDV [65].

The same strategy was exploited to develop a vaccine candidate against the pig pathogen *Actinobacillus pleuropneumoniae*. For vaccine formulation, the antigen ApxIIPF (the pore-forming region of the exotoxin ApxII) was combined with FliC (a.a. 1–99), either by genetic fusion (Figure 7) or by simple mixing. Immune profiling revealed that truncated FliC boosted both humoral (antibody production) and cellular (T-cell activation and cytokine secretion) immune responses in both cases. In challenge trials, truncated FliC improved vaccine protection to 60–80%, compared to just 20% in the antigen-only group. Notably, even without traditional adjuvants (e.g., mineral salts or oil emulsions), genetically fused truncated FliC still provided strong immune enhancement [66].

## 6. Flagellin-Based Peptides Incorporated into Virus-like Particles

A notable feature of viruses is the ability of their capsid proteins to self-assembly into virus-like particles (VLPs) that mimic viruses in terms of size, structure, and the ability to induce potent humoral and cellular immune responses [67]. Chimeric VLPs can be used to display multiple copies of foreign peptide antigens on their surface, enhancing their immunogenicity. To further stimulate the immune response against the presented antigens, flagellin or its derivatives can be incorporated into the VLPs.

The truncated *S. typhimurium* FliC flagellin (a.a. 1–99) was used in the development of a candidate vaccine against foot-and-mouth disease (FMD). The VP1 protein of FMD virus was linked to the N-terminus of i301, a self−assembling protein designed on the basis of the 2-ketone-3-deoxy-6-phosphoglucolic-acid (KDPG) aldolase from a hyperthermophilic archaeon that forms a high-order hyperstable dodecahedron nanocage with 60-mer through five mutations at the interface of the wild−type protein trimer. An additional fusion construct was FliC (a.a. 1–99) linked to the C-terminus of i301. Fusion proteins VP1-i301-FliC (a.a. 1–99) and VP1-i301 were capable of self−assembling into homogeneous nanoparticles of 25–30 nm in size. These fusion proteins induced high−level cytokines production in guinea pigs compared to monomeric VP1 protein and provided clinical protection against viral infection comparable to an inactivated vaccine. Notably, VP1-i301-FliC (a.a. 1–99) particles exerted a stronger stimulating effect compared with VP1-i301, indicating that truncated FliC effectively stimulates TLR5 signal transduction and further enhances immune protection [68].

Another approach exploited a short flagellin-derived peptide, pFlg (a.a. 85–111, INNNLQRVRELAVQSANSTNSQSDLDS), containing a critical TLR5 binding hotspot. The pFlg peptide was linked to the C-terminus of the capsid protein of porcine circovirus 2 (PCV2), which self-assembles into virus-like particles. Immunization elicited enhanced humoral and cellular immune responses along with reduced viremia following challenge compared to pFlg-free protein. These findings indicate that pFlg may potentiate activation of immune response, likely through TLR5 engagement on dendritic cells. However, direct evidence of pFlg-mediated activation of TLR5 signaling pathways remains to be established [69].

**Figure 8:**
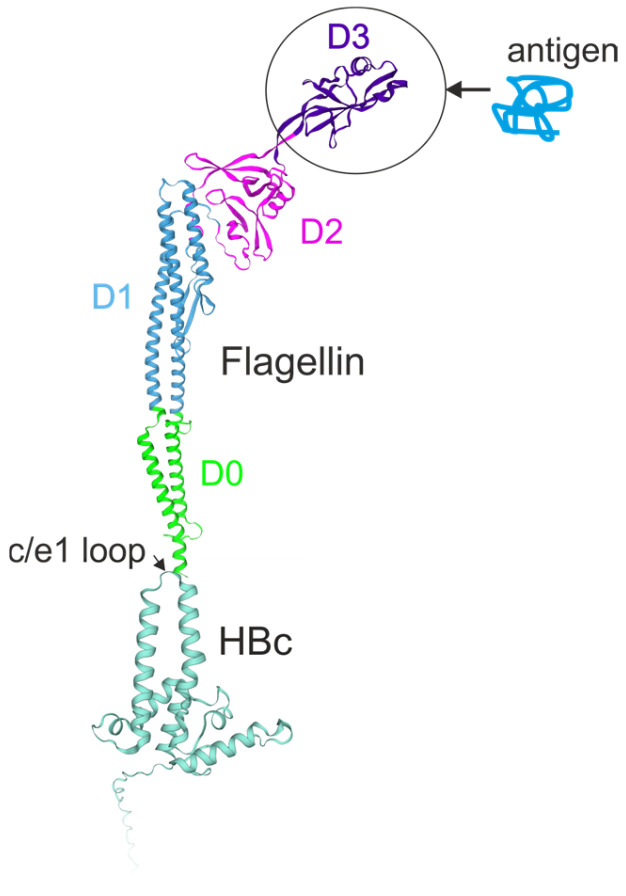
Flagellin/virus-like particle hybrid antigen presentation platform based on HBc particles. The structure of a hybrid protein in which flagellin is inserted into the c/e1 loop region of the HBc antigen is shown. Antigen may be incorporated in place of the D3 domain of flagellin.

Flagellin/virus-like particle hybrid platform has been developed based on Hepatitis B core (HBc) particles and flagellin (FljB) (Figure 8). The flagellin sequence was inserted into the major immunodominant c/e1 loop of HBc, exposed on the surface of HBc particles. The heterologous antigens could be presented by replacing the surface-exposed D3 domain of flagellin (a.a. 192–292) (Figure 8). The M2e peptide of influenza A virus and ovalbumin (OVA) were used as model antigens confirming the feasibility of this platform [70].

## 7. Limitations and Prospects of Flagellin-Adjuvanted Vaccines

Full-length flagellin and its derivatives have demonstrated adjuvant activity in multiple infection models [30]. The proven effectiveness of flagellin-based delivery has enabled several flagellin-fusion vaccine candidates to advance into clinical trials [15]. However, these studies also revealed a number of problems associated with both the production of flagellin fusions and the negative side effects of their use as vaccines.

### 7.1. Structural Instability

One of the critical limitations of the use of flagellin is its inherent structural instability, particularly of its monomeric form. The protein’s functional domains (especially the D0 region) are prone to proteolytic degradation, which compromises its integrity during production and storage [71]. Flagellin (FliC) could be successfully expressed using a cell-free protein synthesis system but accumulated as a C-terminally truncated form (~47 kDa vs. 52.7 kDa) due to proteolytic cleavage at the R453 site in the structurally flexible D0 domain. The presence of an intact D0 domain is of key importance for the adjuvant activity of flagellin, so it cannot be deleted [39]. Neither alanine scanning nor targeted mutagenesis of potential cleavage sites prevented proteolysis, suggesting that the unstructured nature of the D0 domain in monomeric flagellin makes it inherently susceptible to degradation. This structural flexibility appears to be essential for the natural polymerization function of flagellin [72]. Flagellin stability can be increased by creating disulfide bonds in the D0 domain, mimicking natural polymerization stabilization. For example, six cysteine-substituted variants at key positions were created: SS1 (L12C-N484C), SS2 (Q22C-Q473C), SS3 (Q22C-T477C), SS4 (G35C-S457C), SS5 (L36C-D456C), and SS6 (A51C-R451C). Mutants SS5 and SS6 exhibited minimal proteolytic truncation [72].

### 7.2. The Choice of Antigen and Strategy for Its Fusion with Flagellin

The properties of the antigen used in a vaccine, as well as the site of its attachment to flagellin, can unpredictably affect both the immunogenicity and safety profile of the vaccine. The choice of antigen is critical because its properties, such as molecular structure, stability, and immunodominant epitopes, can determine the strength and specificity of the immune response.

For example, early studies explored various approaches to fusing the influenza A virus HA globular head region to flagellin, including attaching it to the C-terminus (C-term format), replacing the D3 domain of flagellin (R3 format), or using a double insertion approach with one antigen’s copy inserted at the C-terminus and another copy replacing the D3 domain (R3.2x format). Preclinical and clinical studies demonstrated that the R3 and R3.2x formats outperformed the C-term format in terms of immunogenicity and safety.

However, when applied to influenza B vaccines, these formats showed limited efficacy due to weak activation of TLR5, resulting in poor immunogenicity [73]. A key difference between the globular heads of HA of influenza A and B viruses is their isoelectric points (pI)—the HA1 subunit of influenza B virus has an unusually high pI (above 9.0), whereas most HA1 domains of influenza A virus have a lower pI value. It was hypothesized that the positively charged influenza B HA head might electrostatically interact with flagellin at neutral pH, potentially disrupting TLR5 signaling or antigen presentation. The R3 format was modified by adding two negatively charged residues to the HA-flagellin linker to reduce charge interactions, and an alternative construct (D3Ins) was created in which HA1 was inserted into the D3 domain rather than replacing it entirely, positioning it further away from the TLR5 binding regions in the D1 domain. It was shown that the introduction of two negatively charged residues moderately improved antibody titers, whereas the use of the D3Ins format provided the most pronounced enhancement in anti-HA1 antibody titers [73]. Thus, the choice of antigen and its fusion strategy play a decisive role in the effectiveness of the vaccine.

### 7.3. Flagellin’s Own Immunogenicity and Associated Adverse Effects

The progression of flagellin-based vaccines through clinical trials and toward commercialization has been limited by reactogenicity of flagellin and substantial adverse effects [74].

In initial Phase 1 trials for seasonal influenza, the flagellin-adjuvanted seasonal influenza vaccine VAX102 (Figure 3), was evaluated. This vaccine candidate was obtained by fusing four copies of the M2e peptide to the C-terminus of flagellin and was co-administered with trivalent inactivated influenza vaccine. These studies documented mild to moderate adverse events, including headache, fatigue, and myalgia, yet overall tolerability was considered acceptable [36].

A subsequent Phase 1 investigation of the VAX102 vaccine alone reported severe adverse effects at higher doses (3 μg and 10 μg), which were linked to increased cytokine and C-reactive protein levels [74]. A further study with a quadrivalent influenza vaccine incorporating VAX102 also revealed severe symptoms in a small number of subjects at elevated doses, although the vaccine was generally well tolerated [41]. The failure of these vaccines to advance to later-stage clinical trials is likely due to the minor to moderate clinical events recorded in the study populations. Consequently, risks associated with cytokine release and antibody responses against flagellin itself represented important obstacles to the clinical development and market approval of this vaccine.

Most vaccines require booster doses to achieve sufficient protection. Repeated exposure to the same antigen stimulates immune memory and enhances the immune response. Flagellin itself is a potent antigen, independent of its TLR5-mediated immunomodulatory effects. Repeated administration would also elicit a stronger immune response to flagellin and diminish the adjuvant’s ability to enhance immune response against target antigen. Moreover, flagellin is a highly conserved protein, present in the flagella of many bacteria that constitute the natural microbiota of healthy humans. Such widespread natural exposure means that peoples may already have pre-existing immunity to flagellin before vaccination even begins. Consequently, upon the first administration of a flagellin-based vaccine, the immune system may mount a rapid, secondary response against the adjuvant itself, potentially compromising the primary response to the target antigen and increasing the risk of reactogenicity.

Therefore, a rational strategy to overcome this challenge is the development of so-called deimmunized flagellin derivatives, engineered to eliminate undesirable immunogenicity while preserving their TLR5-mediated immunomodulatory activity [4]. The main approach to solving this problem is the construction of different truncated forms of flagellin with deletion of the central variable domain or the use of short flagellin-based peptides.

Numerous studies were focused on flagellin derivatives lacking the D3 domain. This approach has been successfully implemented in several preclinical and clinical vaccine candidates, including the VAX128 influenza vaccine. Systematic domain analysis identified the D2/D3-deleted flagellin scaffold as the gold standard for flagellin-adjuvanted recombinant vaccines. Over 60% of published flagellin-based fusion constructs utilize this architecture, demonstrating their efficacy in combating bacterial pathogens, viral and parasitic diseases. However, approximately 70–85% of D2/D3-deleted flagellin fusions form inclusion bodies in bacterial expression systems, requiring denaturation-refolding to restore functional protein. The TLR5-binding activity of these fusion proteins was restored after refolding.

Several studies have explored minimized flagellin constructs (N-terminal D0/D1 domains) that retain TLR5 agonist functionality while eliminating potentially immunodominant variable regions, and even ultra-short flagellin derivatives that retain key PAMP recognition motifs while offering improved pharmaceutical properties for clinical formulations. However, the adjuvant activity of such short peptides may be inferior to that of full-length flagellin.

An alternative approach is to use flagellins from other bacteria. *Bacillus subtilis* flagellin (Hag) shows high sequence similarity to *Salmonella* FljB in the TLR5-binding domains D0 and D1, whereas the off-target response-associated domains D2 and D3 are absent. Thus, Hag can be used as a potent adjuvant with reduced proinflammatory properties [75].

Engineered or naturally occurred deimmunized flagellins can overcome key barriers, associated with flagellin’s own immunogenicity and enable the development of marketable flagellin-based drugs.

## 8. Concluding Remarks

Flagellin is widely used in vaccine development as an immunostimulatory adjuvant. In its adjuvant capacity, flagellin triggers TLR5-dependent innate immune activation, potentiating robust adaptive responses to co-delivered antigens, especially in mucosal vaccination strategies. Concurrently, its carrier function enables linking of epitopes, converting these poorly immunogenic compounds into targets for the production of specific antibodies. As clinical development of flagellin advances, its multifunctional immunomodulatory capacity demonstrates potential for innovative vaccine and therapeutic applications.

Current challenges in the use of flagellin as a vaccine adjuvant include optimization of the flagellin–antigen fusions architecture (deletions of particular domains in flagellin, use of linker sequences, multi-epitope fusion constructs, etc.), addressing pre-existing immunity, and scaling up the production of fusion proteins in accordance with good manufacturing practice requirements.

However, as of the end of 2025, there is no commercially available recombinant vaccine that uses flagellin as an adjuvant. For a flagellin-based vaccine to be viable for widespread clinical use, concerns regarding its reactogenicity and the impact of pre-existing immunity must be thoroughly investigated and definitively resolved. “Balancing harm and harmony” is an essential requirement for progress in this field [76].

## Data Availability

No new data were created or analyzed in this study. Data sharing is not applicable to this article.

## Supplementary Materials

The following supporting information can be downloaded at https://www.mdpi.com/article/10.3390/ijms262110295/s1.
